# The amount of calcifications in pseudoxanthoma elasticum patients is underestimated in computed tomographic imaging; a post-mortem correlation of histological and computed tomographic findings in two cases

**DOI:** 10.1007/s13244-018-0621-6

**Published:** 2018-06-01

**Authors:** Annelotte Vos, Guido Kranenburg, Pim A. de Jong, Willem P. T. M. Mali, Wim Van Hecke, Ronald L. A. W. Bleys, Ivana Isgum, Aryan Vink, Wilko Spiering

**Affiliations:** 1 Department of Pathology, University Medical Center, Utrecht University, Utrecht, The Netherlands; 2 Department of Vascular Medicine, University Medical Center, Utrecht University, PO Box 85500, 3508 GA Utrecht, The Netherlands; 3 Department of Radiology, University Medical Center, Utrecht University, Utrecht, The Netherlands; 4 Department of Anatomy, University Medical Center, Utrecht University, Utrecht, The Netherlands; 5 Image Sciences Institute, University Medical Center, Utrecht University, Utrecht, The Netherlands

**Keywords:** Pseudoxanthoma elasticum, Autopsy, Histology, Radiology, Vascular calcification

## Abstract

**Objectives:**

Pseudoxanthoma elasticum (PXE) is a rare genetic disorder, characterised by elastic fibre degeneration and calcifications in multiple organ systems. Computed tomography (CT) imaging is a potential method to monitor disease progression in PXE patients; however, this method has not been validated. The aim of this study was to correlate histological and computed tomographic findings in PXE patients to investigate the ability of CT scanning to detect these alterations.

**Methods:**

Post mortem total body CT scans were obtained from two PXE patients (a 69-year-old male and 77-year-old female). Autopsy was performed, and 38 tissue samples of the first and 45 tissue samples of the second patient were extensively investigated histologically. The findings were compared with the CT scans.

**Results:**

Degenerated and calcified elastic fibres and calcifications were histologically found in the skin, subcutaneous fat, heart, arteries and pleura and around the oesophagus. On CT imaging only the intradermal alterations of the skin and the larger vascular calcifications were detected. The smaller PXE-related abnormalities were not visible on CT.

**Conclusions:**

With CT imaging vascular calcifications and skin alterations can be monitored in PXE patients. However, many of the subtle PXE-related abnormalities found in other organ systems during the autopsy were not visualised by CT scans. Furthermore, we extended the current knowledge on the disease location of PXE with subcutaneous, oesophageal and pleural lesions.

**Teaching Points:**

*• CT can be used to monitor gross vascular calcifications in PXE patients.*

*• Many subtle PXE-related abnormalities are not visualised by CT scans.*

*• PXE-related alterations can also be found in oesophagus, pleura and subcutaneous fat.*

**Electronic supplementary material:**

The online version of this article (10.1007/s13244-018-0621-6) contains supplementary material, which is available to authorized users.

## Introduction

Pseudoxanthoma elasticum (PXE) or Grönblad-Strandberg syndrome is a rare autosomal recessive disorder characterised by ectopic calcifications of connective tissues [1]. The disease is, in the majority of cases, caused by mutations in the *ABCC6* gene [2–4]. These *ABCC6* gene mutations result in lower levels of inorganic pyrophosphate leading to progressive calcification throughout the body [5].

Previous studies have shown that alterations can be found both histologically and radiologically in the skin, testis and blood vessels [6–10]. Furthermore, histological alterations have been described in the eyes and brain [9–11]. Using imaging techniques, calcifications have been described juxta-articular, in the soft tissues of the extremities and in the breast [6, 8].

The combination of histology and radiology may provide insight into the extent to which the imaging findings may be able to identify the PXE alterations in vivo, important knowledge for further diagnostics and research in living patients. Although some histological and radiological studies have been performed in PXE patients, a correlation study between the two is lacking. The aim of this study was to investigate to what extent histologically determined alterations can be detected with computed tomography (CT) scan in PXE patients.

## Methods

Both tissue samples and CT images were obtained from two autopsy patients (a 69-year-old male and 77-year-old female) diagnosed with PXE ante mortem. One of the patients donated her body to science via the Department of Anatomy of the University Medical Centre Utrecht. From this patient, written informed consent regarding the use of her body for educational and research purposes was obtained during life. For the other patient, relatives gave consent to the post mortem investigations. Collection of the material was approved by the local biobank review committee under protocol number 15–252.

### Radiology

Subjects were scanned post mortem on a Philips Brilliance 256-slice CT scanner (Philips Healthcare, Best, The Netherlands). Tube voltage was 140 kV and tube current 200 mAs. Non-contrast-enhanced CT scans with 0.9-mm slice thickness were acquired. The CT scans were evaluated by one radiologist (PdJ) with 14 years of experience in reading CT scans. The radiologist was blinded to the autopsy results, but aware of the locations that were histologically examined.

### Histology

Tissue samples from the skin, heart, arteries, digestive system, respiratory system, genitourinary system, haematopoietic system, endocrine system and central nervous system (online supplemental Table 1) were obtained the during autopsy and fixed in 4% formaldehyde. The macroscopically calcified samples were subsequently decalcified using diaminoethylene tetraacetic acid solution (EDTA). Decalcification was necessary to maintain the morphology of the tissue specimens. Since histological evaluation of calcification is based on visualisation of the previously altered matrix by the calcification process, and not the calcium ions themselves, decalcification does not influence analysis [12]. Four-micrometre slides were cut and stained with haematoxylin and eosin and, in anatomic locations where elastic fibres were expected, additional elastin van Gieson stain was used. In non-decalcified tissue with altered elastic fibres, a von Kossa stain was used to detect calcifications.

## Results

### Skin

**Fig. 1:**
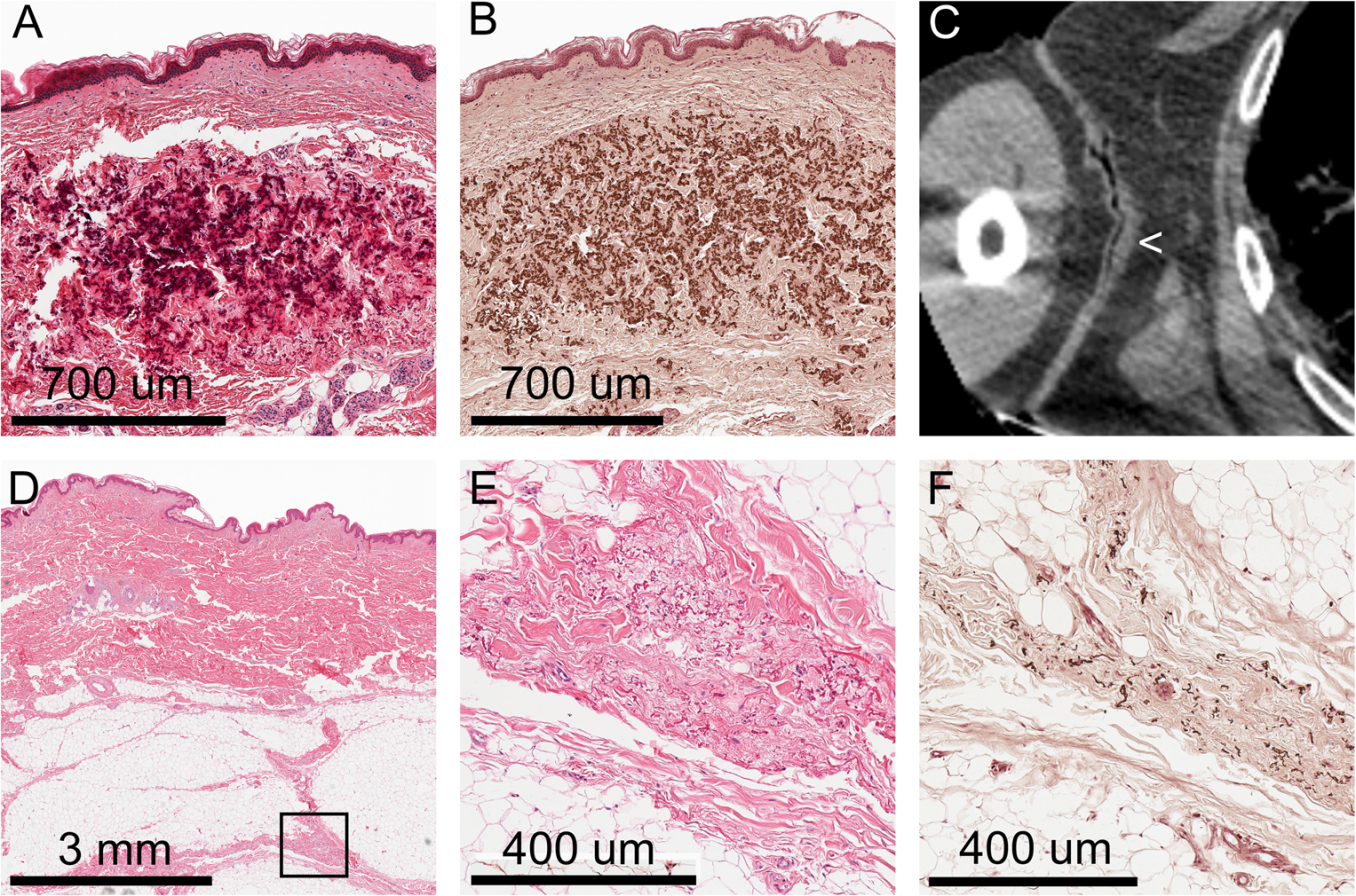
Skin alterations in the pseudoxanthoma elasticum. A–C: In the axillae typical pseudoxanthoma elasticum lesions were found, consisting of clumps of degenerated elastic fibres in the mid and lower dermis (A). Von Kossa stain showed calcifications of these elastic fibres (B). On the CT scan, thickened skin (<) was seen (C). D–F: Other localisations of the skin, macroscopically unremarkable, showed degenerated elastic fibres in the septa of the subcutaneous fatty layer. The marked area in D is shown in E (H&E stain) and F (von Kossa stain)

Classical PXE skin alterations were found at the axillae of both patients, a localisation typically known for skin changes in PXE. Microscopically dense clumps of degenerated and calcified elastic fibres were found in the mid and lower dermis. On CT, these alterations were seen as thickened skin without obvious calcifications. Microscopy of the macroscopically normal skin of the abdomen and extremities showed similar changes that were located in the connective tissue septa between the subcutaneous fatty layer (Fig. 1). On CT, however, no abnormalities were seen at these locations (Table 1).

**Table 1:** Alterations found histologically and radiologically in the two PXE patients

	Histology	Radiology
Macroscopically altered skin	Degeneration and calcification of the elastic fibres in the mid and lower dermis	Thickened skin
Macroscopically normal skin	Degeneration and calcification of the elastic fibres in the septa between the subcutaneous fatty layer	–
Heart	Degeneration and calcification of elastic fibres mainly underneath the endocardial layer and to a lesser extent in fibrous tissue between the cardiomyocytes	–
Arteries (lower extremity, gastroepiploic artery)	Both atherosclerotic intimal lesions and calcifications in the medial layer	More or less circumferential calcifications in case of medial calcification, thick dots of calcification in case of intimal calcification
Arteries (other)	Small scattered calcified elastic fibres in the media and/or internal and external elastic lamina Atherosclerotic intimal lesions	Thick dots of calcification in case of intimal calcification
Central nervous system	Lacunar infarction	–
Central nervous system	White matter abnormalities	Non-specific abnormalities in the white matter area
Central nervous system	Calcification of the small arteries in the area of the globus pallidus and hippocampal area	–
Kidney	Kidney stone	Kidney stone
Adrenal gland	Myelolipoma with calcifications and bony transformation	Calcified adrenal gland
Gallbladder	Gallstones	Gallstones
Lung	Some degenerated and calcified elastic fibres in the pleura	Thickened pleura
Oesophagus	Some degenerated and calcified elastic fibres around the oesophagus	–

### Cardiovascular system

**Fig. 2:**
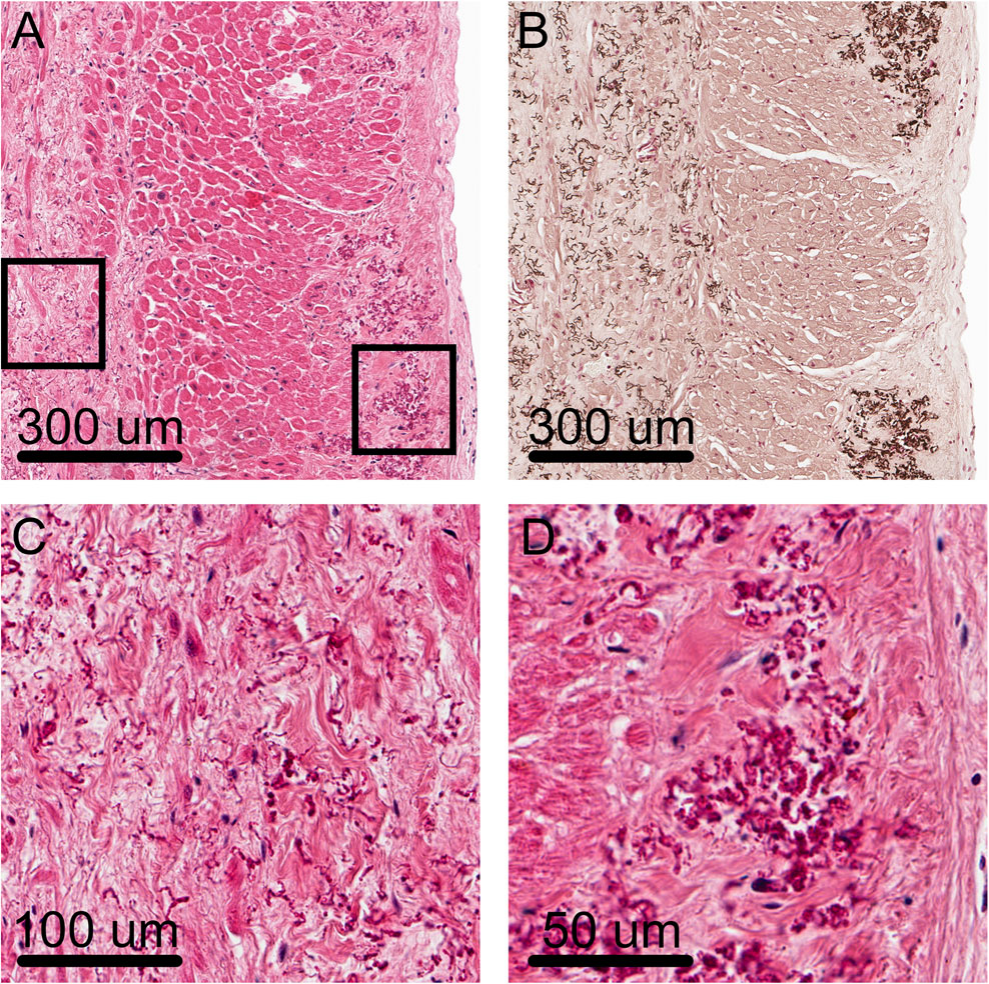
Elastic fibre alterations in the heart. **a** Degenerated and calcified fibres were mainly found below the endocardial layer (right side of the picture). Furthermore, similar fibres were seen in the interstitial fibrous tissue in the myocardium (left side of the picture). **b** Von Kossa stain showing the calcified elastic fibres. **c** Enlarged picture of the abnormal elastic fibres within the interstitial fibrotic tissue in the myocardium. **d** Enlarged picture of the abnormal elastic fibres in the subendocardial layer

The hearts of both patients showed localised degenerated and calcified elastic fibres in the area underneath the endocardial layer, mainly present in both atria. Furthermore, some similar elastic fibres were present in the interstitial fibrous tissue between the cardiomyocytes (Fig. 2). On CT no calcifications were seen in the endo- or myocardial tissue. Calcification of the valves was not present in these patients (Table 1).

**Fig. 3:**
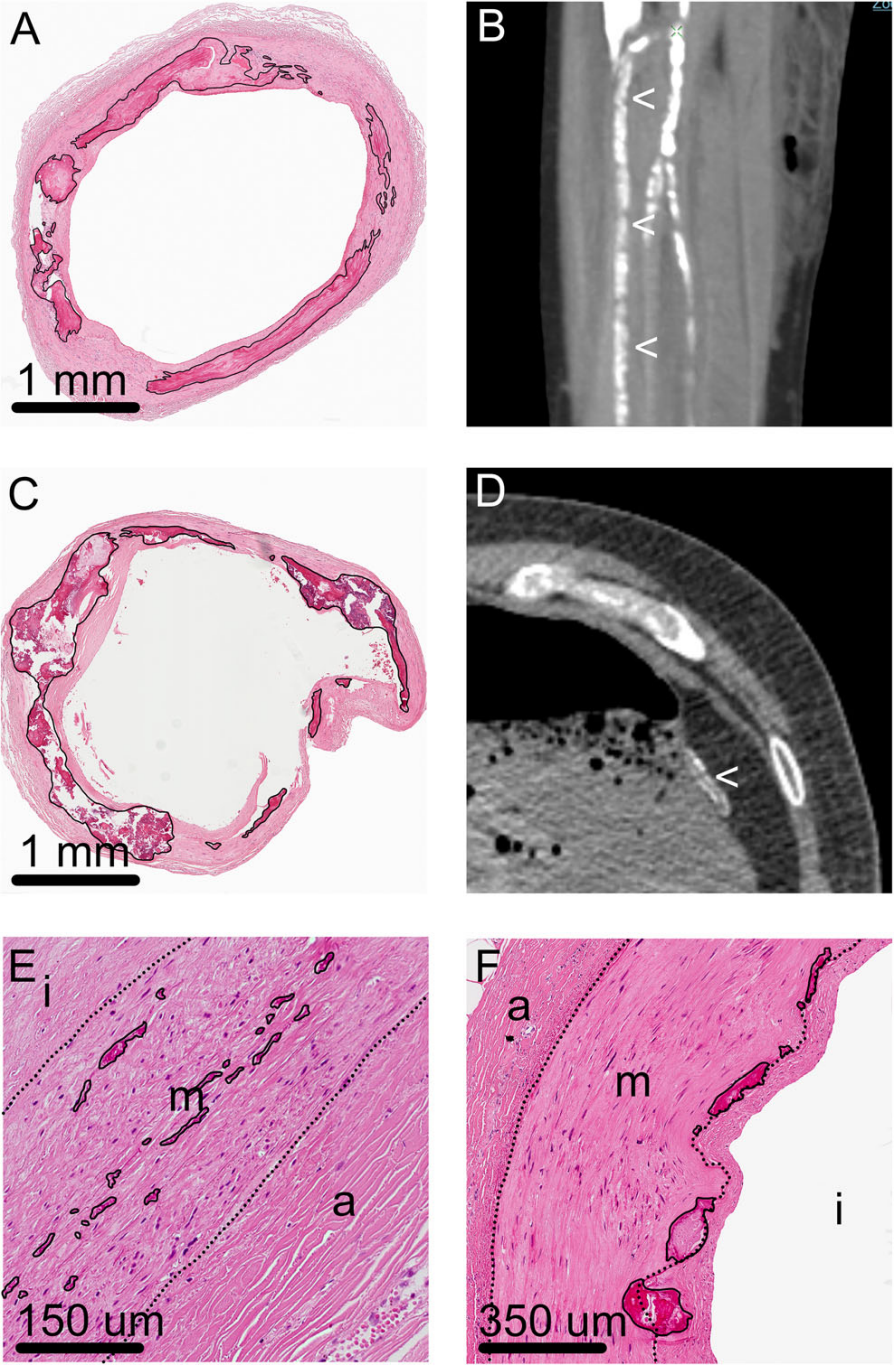
Vascular calcifications. A and B: Extensive calcifications, on the CT scan seen as more or less circumferential calcifications in a longer segment of the vascular wall, were present around the internal elastic lamina and in the media of the vessels of the lower extremities [here the anterior tibial artery (<)]; calcifications are marked with a black line in A. C and D. The same internal elastic lamina and medial calcifications were visible in the gastroepiploic artery, located along the greater curvature of the stomach, in both the histology (calcifications are marked in C) and CT scan (<). E and F: In most of the other large- and middle-sized arteries, variable amounts of calcified elastic fibres were seen in the media (in elastic arteries) and/or around the internal and external elastic lamina (marked). These small calcifications could not be detected on CT scans, on which also many atherosclerotic intimal calcifications were visible (i = intima, m = media, a = adventitia; dotted lines indicate internal and external elastic lamina)

The vascular system showed the presence of both atherosclerotic intimal lesions with calcifications and calcifications in the medial layer and/or around the internal and external elastic lamina of the arterial system. In the lower extremities, large amounts of medial/elastic lamina calcification were present, accompanied by intimal lesions of variable severity. Also the gastroepiploic artery showed extensive calcification of the medial layer and around the elastic lamina. On CT, these medial calcifications were detected as more or less circumferential and present over a longer track of the vascular wall. The other large and middle-sized arteries showed variable amounts of calcified elastic fibres in the media (in elastic arteries) and/or the internal and external elastic lamina (Fig. 3). These small scattered calcified fibres were not detected on CT. Furthermore, in some of the small arteries in the organs (heart, lung, kidney, stomach, pancreas, thyroid), more or less circumferential calcification of the internal elastic lamina was present. These very small vessels were not detected on CT. Besides medial/elastic lamina calcification, also many calcifications were present in the atherosclerotic lesions found in both patients. These calcifications were much more clumped together and therefore, if large enough, detectable on CT (Table 1).

### Central nervous system

**Fig. 4:**
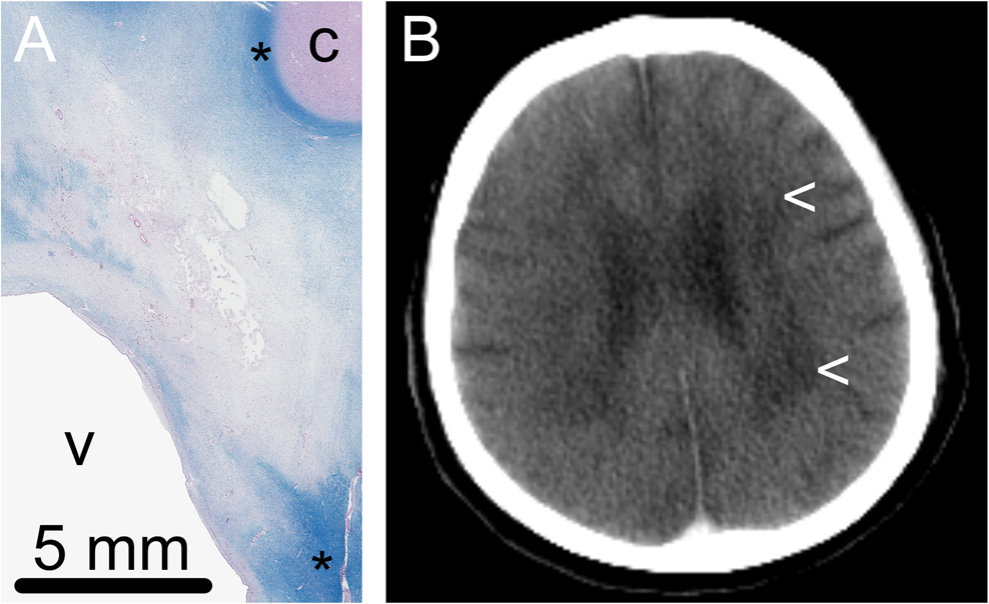
Cerebral white matter lesions. **a** Histological slides (Luxol fast blue-Pas stain) showed dispersion of the matrix with extensive demyelination in the central white matter. Normal myelination is seen subcortically and in the area around the basal nuclei (*). c = cortex, v = ventricle. **b** The CT scan of the patient showed nonspecific white matter abnormalities (<)

A lacunar infarction was found in both patients (one in the left frontal lobe and one in the area of the basal nuclei). Furthermore, in one patient the central white matter showed dispersion of the fibrillary matrix with clear demyelination. The subcortical white matter was unremarkable (Fig. 4). The cerebral microvasculature showed sclerosis of the vascular wall, with vascular calcifications in the area of the globus pallidus. In one of the patients, some calcifications were present in the hippocampal region. On the CT, there were nonspecific abnormalities in the white matter area. The small vascular calcifications in the area of the globus pallidus, present in both patients, were not seen on CT. Also the vascular calcifications in the hippocampal area of one of the patients were not seen (Table 1).

### Other findings

In one of the patients, a kidney stone in the right kidney was found, an observation also made via CT. The same patient also showed a calcified right adrenal gland, microscopically fitting with a myelolipoma with, or combined with, extreme calcifications and bony transformation. Furthermore, small gallstones were present in the gallbladder of this patient. These were seen on CT. In the other patient, some degenerated and calcified fibres were found histologically in the pleura and around the oesophagus. The CT showed a thickened pleura. However, since this patient also suffered from a malignancy in the lung, the cause of this thickening was probably localisation of the lung carcinoma and not alterations caused by PXE. No abnormalities were seen around the oesophagus on CT (Table 1).

## Discussion

PXE is a systemic disease characterised by elastic fibre degeneration and calcification in multiple organ systems. The present study combines radiological and histological findings in two PXE patients to investigate which alterations can and which cannot be seen using CT. The study has two important results. First, most of the abnormalities seen on histological slides were not seen on the CT, except for the intradermal skin alterations and part of the vascular calcifications. Second, this study adds to the existing knowledge regarding the abnormalities that can be seen in PXE patients. Degenerated and calcified elastic fibres were not only found in the skin, arteries, heart and pleura, where they have been described before, but also in the fibrous bands in between the subcutaneous fat tissue and around the oesophagus.

### Skin

Besides the histological skin alterations typical for PXE, we also found histological alterations in macroscopically unaltered skin, most abundantly located in the subcutaneous fat. The presence of some abnormal elastic fibres in non-lesional skin has been described before [13]. However, the presence of subcutaneous lesions at these locations has not been described before. A possible explanation could be that most knowledge about histological skin alterations is obtained by studies in skin biopsies, with only small amounts of subcutaneous fat.

### Cardiovascular system

In the heart degenerated and calcified elastic fibres were present subendocardially and in the interstitium between the cardiomyocytes. The presence of these altered elastic fibres in the cardiac tissue has been described before and has been suggested as a cause of restrictive cardiomyopathy and congestive heart failure [9, 14].

The vascular system showed the presence of both atherosclerotic intimal lesions with calcifications and calcifications present in the medial layer and/or around the internal and external elastic lamina of the vascular wall. In case of extensive calcification of the medial layer or elastic lamina, on CT scans a more or less circumferential pattern over a longer segment of the vessel was seen. This pattern is comparable to the pattern of medial calcification seen on X-rays described in the scarce literature [15]. Our findings of vascular calcifications and the combination of both atherosclerosis and medial calcification are consistent with previous findings [16].

### Central nervous system

In both patients, a lacunar infarction was found. The combination of PXE and lacunar infarctions of the brain has been described before as a complication of small vessel disease [17]. Also the white matter lesions, as seen in one of our patients, have been described before in association with PXE [11, 17, 18]. The combination of lacunar infarctions and white matter lesions have been described in association with cognitive deterioration, although reports also mention extensive white matter lesions in a patient with normal baseline cognitive status [17]. On CT scans nonspecific abnormalities could be found in the white matter area. For diagnostic purposes and further research, MRI probably is a better imaging technique.

### Other findings

A kidney stone was found in one of the patients. A possible relation between PXE and nephrolithiasis has been suggested before [19]. However, in most of the patients described phosphocalcic abnormalities were present, which was not the case in our patient. Since nephrolithiasis is not a rare condition, it is not unlikely that this is a coincidental finding. In the same patient, also gallstones and a calcified adrenal gland were found. It is unknown to which extent this can be related to PXE. Furthermore, in the other patient some calcified fibres were found in the pleura and around the oesophagus. To our knowledge, degenerated and calcified elastic fibres have not been described in these locations before.

An important limitation of this study is the limited number of bodies studied, which can be explained by the low incidence of the disease and low autopsy rate in The Netherlands. Due to this small number of patients, it is possible that by chance we selected two patients in which many abnormalities were not seen on CT scans, while in larger series of patients this would not have been the case. Therefore, our findings need confirmation in a larger series of patients. Nevertheless, the findings in our patients during autopsy are comparable to those described in the literature. Furthermore, we did not study the eyes of the patients. However, most of the ocular findings in PXE (peau d’orange, angioid streaks, chorioretinal atrophies) can already be diagnosed in living patients using a variety of diagnostic techniques [20]. It is doubtful whether CT, with a relatively low resolution for a small organ such as the eye, can contribute to these diagnoses.

In conclusion, autopsy of two PXE patients revealed degenerated and calcified elastic fibres and calcifications in skin, heart and arteries, but also in between the subcutaneous fat tissue, in the pleura and around the oesophagus, locations where they have not been described before. Only the intradermal and vascular calcifications were seen on CT. Our results indicate that CT can be used to study vascular calcifications in this patient population. However, while doing so, one should keep in mind that small calcifications are not visible using this technique.

## Electronic supplementary material

ESM 1 (PDF 17 kb)
