# Supplementary material for: The amount of calcifications in pseudoxanthoma elasticum patients is underestimated in computed tomographic imaging; a post-mortem correlation of histological and computed tomographic findings in two cases
Source: Insights Imaging. 2018 Jun 1;9(4):493–8. doi: 10.1007/s13244-018-0621-6 (PMC6108976; doi:10.1007/s13244-018-0621-6)
Supplement: Supplementary file 1 — (PDF 17 kb) [file 13244_2018_621_MOESM1_ESM.pdf]

Online Supplement

This data supplement has been provided by the authors to give the readers additional information about their work.

Supplement to: Vos A, Kranenburg G, De Jong PA, Mali WPTM, Van Hecke W, Bleys RLAW, Isgum I, Vink A, Spiering W. The amount of calcifications in pseudoxanthoma elasticum patients is underestimated in computer tomographic imaging; a post-mortem correlation of histological and computed tomographic findings in two cases.

Table of contents:

Content	Page
Supplemental table I: List of examined tissue samples	2

Supplemental table 1 List of examined tissue samples

Organs	Case 1	Case 2
Skin	Macroscopically affected skin	Macroscopically affected skin
	Macroscopically unaffected skin	Macroscopically unaffected skin
Heart	Ventricular myocardium	Ventricular myocardium
	Atrial myocardium	
	Atrioventricular node	Atrioventricular node
	Pericardium	
Vessels	Abdominal aorta	Abdominal aorta
	Thoracic aorta	Thoracic aorta
	Common carotid artery	Common carotid artery
	Coronary artery	Coronary artery
	Celiac trunk	Celiac trunk
	Splenic artery	Splenic artery
	Superior mesenteric artery	Superior mesenteric artery
	Renal artery	Renal artery
		Inferior mesenteric artery
		Common iliac artery
		Internal iliac artery
	External iliac artery	External iliac artery
	Femoral artery	
		Superficial femoral artery
	Anterior tibial artery	Anterior tibial artery

	Intracranial internal carotid artery<br>Vertebral artery<br>Basilar artery	Intracranial internal carotid artery<br>Anterior cerebral artery<br>Medial cerebral artery<br>Posterior cerebral artery<br>Vertebral artery<br>Basilar artery
Digestive system	Esophagus<br>Stomach<br>Liver	Esophagus<br>Stomach<br>Liver<br>Pancreas
Respiratory system	Lungs with pleura<br>Trachea	Lungs with pleura
Genitourinary system	Bladder<br>Kidney	Testicle<br>Prostate<br>Bladder<br>Kidney
Hematopoietic system	Spleen<br>Bone marrow<br>Lymph node	Spleen<br>Bone marrow<br>Lymph node
Endocrine system	Adrenal<br>Thyroid	Adrenal<br>Thyroid
Central nervous system	Spinal cord<br>Cerebral cortex	Cerebral cortex

	Cerebellum<br><br>Basal nuclei<br><br>Hippocampus<br><br>Brain stem	Cerebellum<br><br>Basal nuclei<br><br>Hippocampus<br><br>Brain stem
